# Altered responsiveness of the antioxidant system in chronically stressed animals: modulation by chronic lurasidone treatment

**DOI:** 10.1007/s00213-022-06140-6

**Published:** 2022-04-23

**Authors:** Vittoria Spero, Maria Serena Paladini, Paola Brivio, Marco Andrea Riva, Francesca Calabrese, Raffaella Molteni

**Affiliations:** 1 Department of Medical Biotechnology and Translational Medicine, University of Milan, Via Vanvitelli 32, 20129 Milan, Italy; 2 Present Address: Department of Physical Therapy and Rehabilitation Science; Brain and Spinal Injury Center, University of California at San Francisco, San Francisco, CA USA; 3 Department of Pharmacological and Biomolecular Sciences, University of Milan, Via Balzaretti 9, 20133 Milan, Italy

**Keywords:** Stress response, Redox balance, Gene expression, Antipsychotic, Rat brain

## Abstract

**Rationale:**

Although the occurrence of stressful events is very common during life, their impact may be different depending on the experience severity and duration. Specifically, acute challenges may trigger adaptive responses and even improve the individual’s performance. However, such a physiological positive coping can only take place if the underlying molecular mechanisms are properly functioning. Indeed, if these systems are compromised by genetic factors or previous adverse conditions, the response set in motion by an acute challenge may be maladaptive and even cause the insurgence or the relapse of stress-related psychiatric disorders.

**Objectives:**

On these bases, we evaluated in the rat brain the role of the antioxidant component of the redox machinery on the acute stress responsiveness and its modulation by potential detrimental or beneficial events.

**Methods:**

The expression of several antioxidant enzymes was assessed in different brain areas of adult male rats exposed to acute stress 3 weeks after a chronic immobilization paradigm with or without a concomitant treatment with the antipsychotic lurasidone.

**Results:**

The acute challenge was able to trigger a marked antioxidant response that, despite the washout period, was impaired by the previous adverse experience and restored by lurasidone in an anatomical-specific manner.

**Conclusions:**

We found that a working antioxidant machinery takes part in acute stress response and may be differentially affected by other experiences. Given the essential role of stress responsiveness in almost every life process, the identification of the underlying mechanisms and their potential pharmacological modulation add further translational value to our data.

## Introduction

A proper stress response is crucial to maintain homeostasis in challenging or potentially detrimental conditions. However, this is not always easily achievable since it depends on the characteristics of the stress itself, as well as of the subject who faces it. For example, stressful events are usually perceived as harmful experiences, but when limited in time and intensity they can even become positive stimuli. Specifically, short and mild challenging events trigger different concerted and dynamic processes aimed at restoring the perturbed homeostatic condition, thus leading to a better performance (McEwen 2017). The beneficial response to acute stress, however, can be impeded by specific conditions such as aging, concomitant illnesses (Yiallouris et al. 2019), or severe or prolonged stress exposure. Chronic stress, in particular, represents not only the main environmental risk factor for the development of psychiatric disorders, but also one of the candidates for their relapse after treatment (Alves et al. 2017; Sheets and Craighead 2014; You and Conner 2009). Indeed, it is now known that exposure to severe adversity can cause long-lasting maladaptive changes in the brain that might contribute to the vulnerability to stress-related psychiatric illnesses (Bondade et al. 2019; Hanford et al. 2019; Koo and Wohleb 2021). In this context, it is of crucial importance to unveil the circuits and systems involved in the long-lasting signature of adverse experiences and how those experiences may affect the molecular response to further challenges.

Furthermore, the responses to acute and prolonged stress are divergent under many aspects, with increased neuronal plasticity (Chowdhury et al. 2000; Molteni et al. 2008), neurotrophic factor synthesis and activity (Shi et al. 2010; Yuen et al. 2009), and enhanced working memory (Brivio et al. 2020a, b; Popoli et al. 2013) after acute stress, while reduced connectivity (Ghosal et al. 2017), neuronal atrophy (Ota et al. 2014), and deficits in cognitive and memory performance (Calabrese et al. 2017; Veena et al. 2009) were observed after chronic stress.

The increased brain activity in response to a stressor, be it acute or chronic, requires more oxygen and energy than in homeostatic conditions. In this context, mitochondria play a vital role, as they are responsible for meeting the energy demand through the oxidative phosphorylation machinery that enables aerobic ATP generation, metabolic pathways, and—especially under stressful situations—the production of most cellular reactive oxygen species (ROS) (Manoli et al. 2007). Additionally, the increased amino acids and neurotransmitters metabolism occurring in these conditions results, once again, in increased ROS production (Hermida-ameijeiras et al. 2004). The susceptibility of brain tissues to oxidative stress, caused by the high oxygen demand and the enrichment in unsaturated fatty acids (Cobley et al. 2018), renders the antioxidant response crucial under stressful conditions. Accordingly, growing evidence correlates the unbalances in oxidative homeostasis and the impaired activity of antioxidant enzymes with the development of psychiatric diseases (Li et al. 2018; Paladini et al. 2021; Rossetti et al. 2020).

On these bases, the aim of the present study was to investigate in the rat brain if and how the effects on antioxidant gene expression exerted by an acute challenge can be altered by a previous chronic stress exposure, with a 3-week washout period in between to mimic a recovery phase, a procedure previously used (Brivio et al. 2020a). Furthermore, we also evaluated the ability of a chronic treatment with the antipsychotic lurasidone to modulate acute stress responsiveness. We chose lurasidone given its capability to ameliorate redox unbalances (Rossetti et al. 2018) and to boost neuroplasticity (Luoni et al. 2014), as well as the HPA axis functionality (Calabrese et al. 2020) also in dynamic conditions (Fumagalli et al. 2012).

More in detail, we assessed the gene expression profile of several enzymes and scavengers with different target substrates and function, namely sulfiredoxin-1 (*Srxn1*), metallothionein-1α (*Mt1α*), glutathione peroxidase-1 (*Gpx1*), and glutathione peroxidase-4 (*Gpx4*), in order to have a broad view of the antioxidant response. Furthermore, these genes are downstream effectors of the antioxidant transcription factor Nrf2 activation (Ma 2013), that our group already demonstrated to be implicated in stress response and to be modulated by chronic treatment with lurasidone (Rossetti et al. 2018) as well as by the antipsychotic blonanserin (Paladini et al. 2021).

The analyses were performed in the dorsal and ventral hippocampus (DH and VH, respectively), as well as in the prefrontal cortex (PFC), i.e., brain regions of crucial importance for both chronic and acute stress response and strongly interconnected with each other (Sigurdsson and Duvarci 2016). In particular, the dorsal hippocampus projections to the prefrontal cortex are responsible for memory consolidation and cognition-related inputs integration, whereas ventral hippocampus projections are responsible for emotional control (Bueno-Junior et al. 2018; Liu and Carter 2018). Furthermore, these areas are strongly implicated in the pathophysiology of several stress-related psychiatric diseases (Krishnan and Nestler 2010; McEwen et al. 2016).

## Materials and methods

### Animals

Adult male Sprague–Dawley rats were purchased from a commercial breeder (Charles River, Calco, Italy) and brought into the animal facility 2 weeks before the beginning of the experiment. The animals were socially housed (*n* = 3/4 per cage) with free access to food and water and maintained on a 12-h dark/light cycle at constant temperature (22 °C) and humidity (50 ± 5%). All procedures used in this study were conducted according to the authorization from the Health Ministry n° 151/2017-PR in full accordance with the Italian legislation on animal experimentation (DL 26/2014) and adherent to EU recommendations (EEC Council Directive 2010/63).

### Stress paradigms and pharmacological treatment

After 2 weeks of habituation to the animal facility, rats were randomly assigned to control (CTRL) or chronic restraint stress (CRS) groups. Control rats were left undisturbed in their home cages, whereas stressed rats were exposed to 2 sessions/day of immobilization in transparent plexiglass cylinders (7.5 × 19 cm, 1 h/session, at random hours to avoid habituation) for 4 weeks. After the first week of the experiment, both control and stressed animals were divided into two subgroups to receive for the subsequent 3 weeks vehicle (1% (w/v) hydroxyethylcellulose, 1 ml/kg) or lurasidone (3 mg/kg Sumitomo Dainippon Pharma Co. Ltd, Japan) administered by oral gavage as previously reported (Rossetti et al. 2018). At the end of the fourth week of the experiment, all the animals underwent a washout period of 3 weeks in which all the experimental manipulations were suspended. After that, half of the animals of each subgroup were randomly selected to be exposed to 1 h of acute immobilization stress (AS), while the other half were left undisturbed in the home cages.

**Fig. 1:**
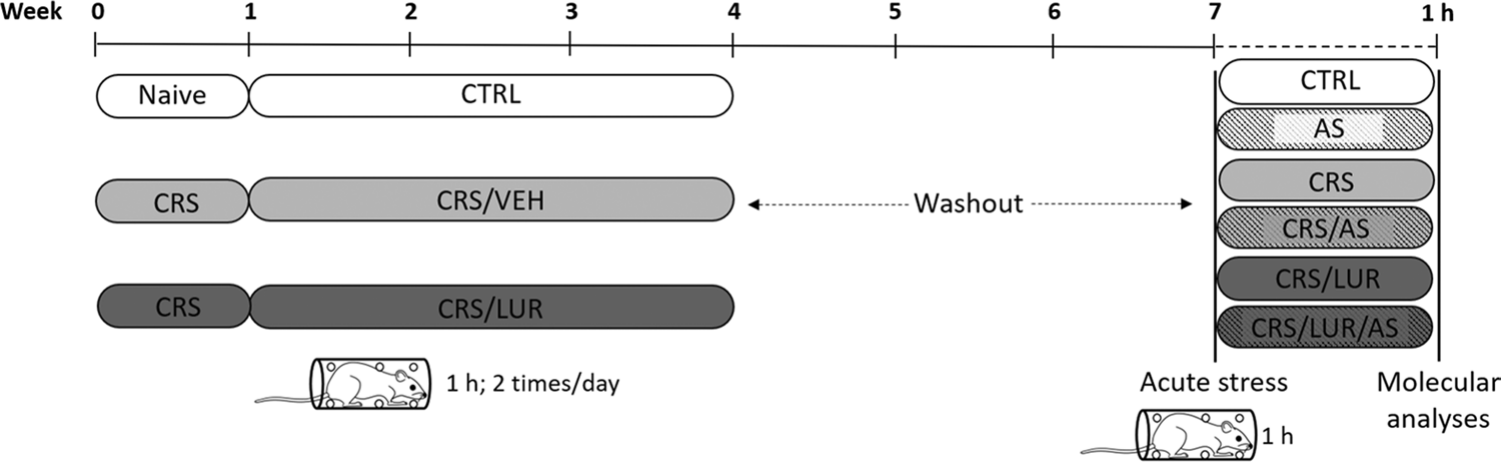
Experimental design. Adult Sprague–Dawley rats were exposed to 4 weeks of chronic restraint stress. After the 1st week of stress exposure, the animals of each group were randomly assigned to saline or lurasidone administration for the following 3 weeks. At the end of the 4th week, the animals underwent a 3-week period of washout, during which they were left undisturbed in their cages. At the end of this period, half of the animals from each group were exposed to a 1-h-long acute session of restraint stress, in order to obtain six experimental groups (*N* = 7–10). All the animals were sacrificed 1 h after the acute stress

Based on this experimental design, depicted in Fig. 1, we obtained the following 6 experimental groups each consisting of 10 animals:

1. Naïve control animals (CTRL), i.e., rats treated with vehicle and not exposed to chronic and/or acute stress;
2. Acutely stressed animals (AS), i.e., rats treated with vehicle and exposed only to the acute stress;
3. Chronically stressed animals (CRS), i.e., rats treated with vehicle and exposed only to chronic restraint stress;
4. Chronically stressed animals exposed to acute stress (CRS/AS), i.e., rats treated with vehicle and exposed to both chronic and acute restraint stress;
5. Chronically stressed animals that received lurasidone (CRS/LUR), i.e., rats treated with lurasidone and exposed only to chronic restraint stress;
6. Chronically and acutely stressed animals that received lurasidone (CRS/LUR/AS), i.e., rats treated with lurasidone and exposed to both chronic and acute restraint stress.

One (1) hour after the acute stress, all the animals were euthanized by decapitation, dorsal hippocampus (DH), ventral hippocampus (VH), and prefrontal cortex (PFC) were rapidly dissected according to Paxinos and Watson atlas coordinates, frozen on dry ice and stored at – 80 °C for further molecular analyses.

### RNA preparation and quantitative real-time PCR analyses

Total RNA was isolated by a single step of guanidinium isothiocyanate/phenol extraction using PureZol RNA isolation reagent (Bio-Rad Laboratories, Italy) according to the manufacturer’s instructions and quantified by spectrophotometric analysis. The samples were then processed for real-time reverse transcription-polymerase chain reaction (RT-PCR) to measure the mRNA levels of *Srxn1* (Rn04337926_g1), *Mt1α* (Rn00821759_g1), *Gpx1* (Rn00577994_g1), and *Gpx4* (Rn00820816_g1) by using Life Technologies gene expression assays. Samples were run in 384 well formats in triplicate as multiplexed reactions with a normalizing internal control (β-actin). Thermal cycling started with incubation at 50 °C for 10 min (RNA retro-transcription) and then at 95 °C for 5 min (TaqMan polymerase activation). After this initial step, 39 cycles of PCR were performed. Each PCR cycle consisted of heating the samples at 95 °C for 10 s to enable the melting process and then for 30 s at 60 °C for the annealing and extension reaction. A comparative cycle threshold (Ct) method was used to calculate the relative target gene expression vs. the control group. Specifically, fold change for each target gene relative to β-actin was determined by the 2^−Δ(ΔCT)^ method, where ΔCT = CT(target) – CT(β-actin) and Δ(ΔCT) = CT(exp. group) – CT (control group), and CT is the threshold cycle. For graphical clarity, the obtained data were then expressed as percentage vs. control group, which has been set at 100%.

### Antioxidant z-activation calculation

As a comprehensive analysis of the impact of our experimental paradigm, we measured the global “antioxidant” z-activation by averaging the normalized score (z-score) of each of the antioxidant genes measured for each animal in all the cerebral areas analyzed. Z-scores are standardized scores (by the group mean and group standard deviation) and no normal assumption is made (Guilloux et al. 2011). They indicate how many standard deviations (σ) an observation (X) is above or below the mean of a control group (μ). In particular, the z-score of the antioxidant response was calculated for each animal using the formula:

$${\mathrm{Z}}_{\mathrm{act}} =\frac{(\frac{\mathrm{X}-\upmu }{\upsigma })Srxn1 +(\frac{\mathrm{X}-\upmu }{\upsigma })Mt1\alpha +(\frac{\mathrm{X}-\upmu }{\upsigma })Gpx1 +(\frac{\mathrm{X}-\upmu }{\upsigma })Gpx4 }{Number of parameters}$$

where X represents the individual gene expression data and μ and σ represent the mean and the standard deviation of the control group.

### Statistical analyses

The impact of chronic stress and lurasidone treatment on acute responsiveness was analyzed by two-way analyses of variance (ANOVA), with acute stress and the previous manipulations as independent variables and the specific gene examined as a dependent variable. Fisher’s protected least significant difference (LSD) test was used for post hoc comparisons of means. All the statistical analyses were performed using GraphPad Prism software. Significance for all tests was assumed for *P* value < 0.05. For graphic clarity, data are presented as percentage versus controls (set at 100%) ± standard error of the mean (SEM). The Z-antioxidant activation is shown as Δ activation of acutely stressed groups compared to the respective—non-exposed to acute stress—controls. The Shapiro–Wilk normality test was performed to analyze the distribution of samples for every experimental group. The molecular analyses were repeated 3 times, and outliers that were more than two standard deviations away from the mean were excluded, as they were likely to be the result of technical errors.

## Results

### Gene expression analyses of antioxidant mediators in rats exposed to an acute challenge after a three-week washout from chronic stress exposure: modulation by lurasidone treatment

To evaluate the responsiveness to the acute challenge and its potential modulation in terms of redox balance, we analyzed the gene expression of different antioxidant enzymes, i.e., *Srxn1*, *Mt1α*, *Gpx1*, and *Gpx4* in three brain regions strongly involved in the stress response, namely the dorsal hippocampus, ventral hippocampus, and prefrontal cortex, whose structure and function are compromised in several stress-related psychiatric disorders. *Srxn1* is a small enzyme that catalyzes the reduction of hyper-oxidized peroxiredoxins (PRXs) in an ATP-dependent manner, allowing them to be reactivated as reducers of H_2_O_2_ in H_2_O (Soriano et al. 2008; Sunico et al. 2016). *Mt1α* is part of a family of small, cysteine-rich, and heavy metal-binding proteins that, besides the classic antioxidant activity, participate in an array of protective stress responses, promoting cell cycle and inhibiting macrophages activation and inflammatory pathways (Penkowa 2006). *Gpx1* is a cytosolic and mitochondrial isoform of glutathione peroxidases, particularly effective in the removal of intracellular hydrogen peroxide, as well as other soluble hydroperoxides released from the membrane lipids (Lubos et al. 2011). Finally, *Gpx4* is specifically involved in the reduction of lipid peroxides, such as the ones produced by iron activity that can cause the so-called ferroptosis, a type of cell death characterized by higher oxidative stress and important mitochondrial damage (Maiorino and Conrad 2018).

#### Dorsal hippocampus

**Fig. 2:**
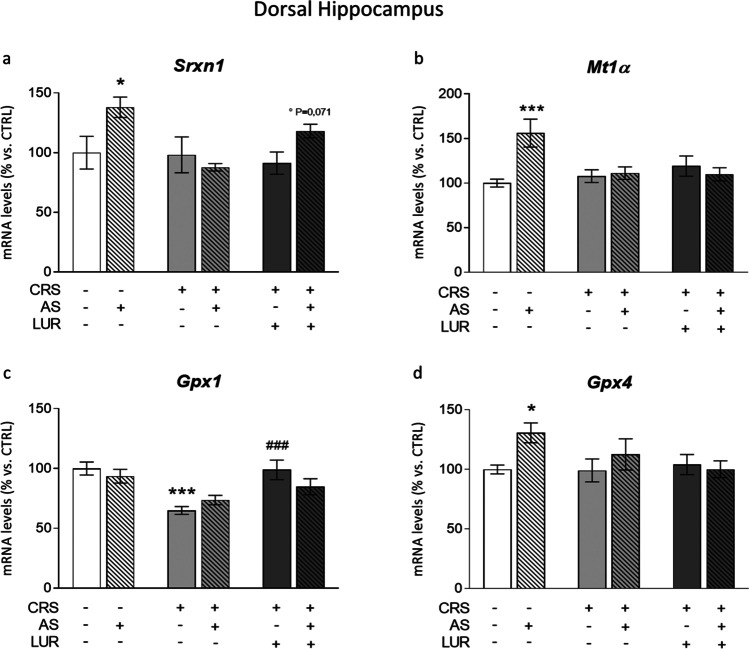
Analysis of antioxidant genes *Srxn1*, *Mt1α*, *Gpx1*, and *Gpx4* levels in the dorsal hippocampus of rats exposed to acute and chronic stress and treated with lurasidone. The mRNA levels of *Srxn1* (**a**), *Mt1α* (**b**), *Gpx1* (**c**), and *Gpx4* (**d**) were analyzed in the dorsal hippocampus of chronically stressed rats, treated with lurasidone and exposed to acute stress after a period of washout. The data, expressed as a percentage of control animals (CTRL, set at 100%), represent the mean ± SEM of at least 7 independent determinations. *P < 0.05; ***P < 0.001 vs. CTRL; ^###^*P* < 0.001 vs. CRS; °*P* < 0,05 vs. CRS/LUR (two-way ANOVA with Fisher’s protected LSD)

One hour after the acute challenge, we observed an overall increase in the mRNA levels of the antioxidant enzymes examined in naïve animals. In particular, the two-way ANOVA indicated a significant effect of AS (*F*_1, 45_ = 4.429; *P* = 0.0409) on *Srxn1*, whose gene expression was increased by the acute stress in control animals (AS: + 38% vs. CTRL, *P* < 0.05; Fig. 2a). This upregulation was not found in rats previously exposed to CRS, but was largely restored following chronic lurasidone treatment (CRS/LUR/AS: + 34% vs. CRS/LUR, *P* = 0.0705).

The analysis of *Mt1α* expression revealed a significant AS effect (*F*_1, 49_ = 4.221; *P* = 0.0453). Indeed, acute stress increased the expression of this enzyme in naïve rats (AS: + 56% vs. CTRL, *P* < 0,001, Fig. 2b) but not in CRS rats, independently from the pharmacological treatment.

With respect to *Gpx1*, no acute stress modulation was detected by two-way ANOVA whereas a significant effect of the previous conditions was observed (*F*_2, 42_ = 11.26; *P* = 0.0001). Specifically, *Gpx1* mRNA levels were reduced in chronically stressed animals (CRS: − 35% vs. CTRL, *P* < 0.001, Fig. 2c). Noteworthy, lurasidone was able to counteract the CRS-induced downregulation thus maintaining *Gpx1* expression similar to the CTRL group (CRS/LUR: + 52% vs. CRS, *P* < 0.001).

Lastly, an almost significant AS effect (*F*_1, 48_ = 3.519; *P* = 0.0667) was found by two-way ANOVA for *Gpx4*, in line with the increased mRNA levels observed after the acute challenge only in control animals (AS: + 31% vs. CTRL, *P* < 0.05, Fig. 2d). Conversely, no changes were found in the other experimental groups.

#### Ventral hippocampus

**Fig. 3:**
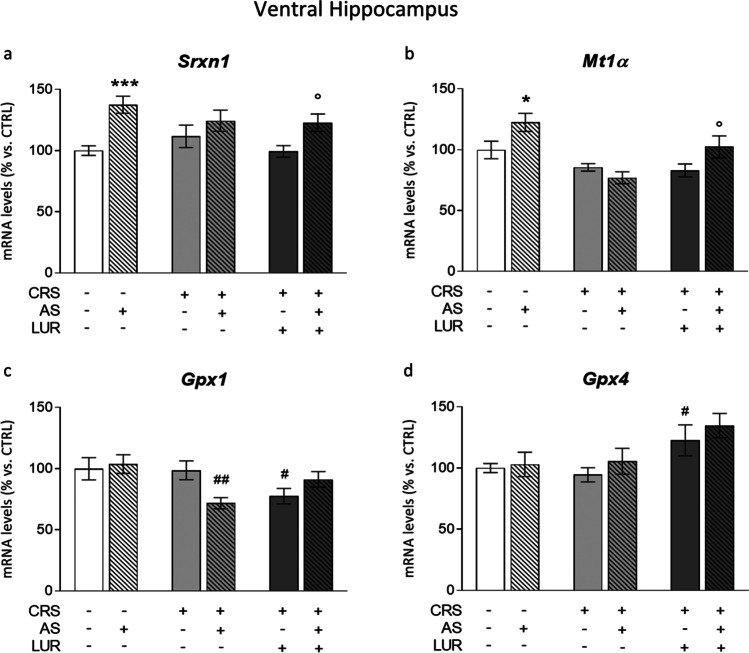
Analysis of antioxidant genes *Srxn1*, *Mt1α*, *Gpx1*, and *Gpx4* levels in the ventral hippocampus of rats exposed to acute and chronic stress and treated with lurasidone. The mRNA levels of *Srxn1* (**a**), *Mt1α* (**b**), *Gpx1* (**c**), and *Gpx4* (**d**) were analyzed in the ventral hippocampus of chronically stressed rats, treated with lurasidone and exposed to acute stress after a period of washout. The data, expressed as a percentage of control animals (CTRL, set at 100%), represent the mean ± SEM of at least 8 independent determinations. *P < 0.05; ***P < 0.001 vs. CTRL; ^#^*P* < 0.05; ^##^*P* < 0.01 vs. CRS; °*P* < 0.05 vs. CRS/LUR (two-way ANOVA with Fisher’s protected LSD)

In the ventral region of the hippocampus, the two-way ANOVA analysis indicated a significant main effect of AS on *Srxn1* expression (*F*_1, 51_ = 18.28; *P* < 0.0001), which was significantly increased by the acute challenge in the naïve group (AS: + 38% vs. CTRL, *P* < 0.001, Fig. 3a). This AS-induced upregulation was not observed in animals previously exposed to chronic stress, whereas it was preserved in animals exposed to both chronic stress and lurasidone treatment (CRS/LUR/AS: + 24% vs. CRS/LUR, P < 0.05).

Regarding *Mt1α*, we found a significant AS effect (*F*_1, 54_ = 4,471; *P* = 0.0391), as well as an effect of the previous conditions (*F*_2, 54_ = 10.92; *P* = 0.0001). More in detail, the acute stress increased *Mt1α* mRNA levels in naïve animals (AS: + 22% vs. CTRL, *P* < 0.05, Fig. 3b) but not in rats previously exposed to CRS. Interestingly, chronic lurasidone treatment in CRS rats was able to normalize the upregulation of *Mt1α* following the acute challenge (CRS/LUR/AS: + 23% vs. CRS/LUR; *P* < 0.05).

On the contrary, acute stress per se did not modulate *Gpx1* expression; nevertheless, we observed a significant two-way ANOVA effect of the previous conditions (*F*_2, 52_ = 3.805; *P* = 0.0287). Indeed, reduced *Gpx1* mRNA levels were specifically found in acutely stressed rats previously exposed to chronic stress (AS/CRS: − 27% vs. CRS, *P* < 0.01, Fig. 3c). Furthermore, despite lurasidone downregulated the enzyme levels (CRS/LUR: − 21% vs. CRS, *P* < 0.05), it produced a small increase in *Gpx1* expression in the CRS/LUR/AS group.

Concerning *Gpx4*, we found a two-way ANOVA significant effect of the previous conditions (*F*_2, 51_ = 5.999; *P* = 0.0046), with increased levels of this enzyme only in animals exposed to chronic stress and treated with lurasidone (CRS/LUR: + 29% vs. CRS, *P* < 0.05; Fig. 3d).

#### Prefrontal cortex

**Fig. 4:**
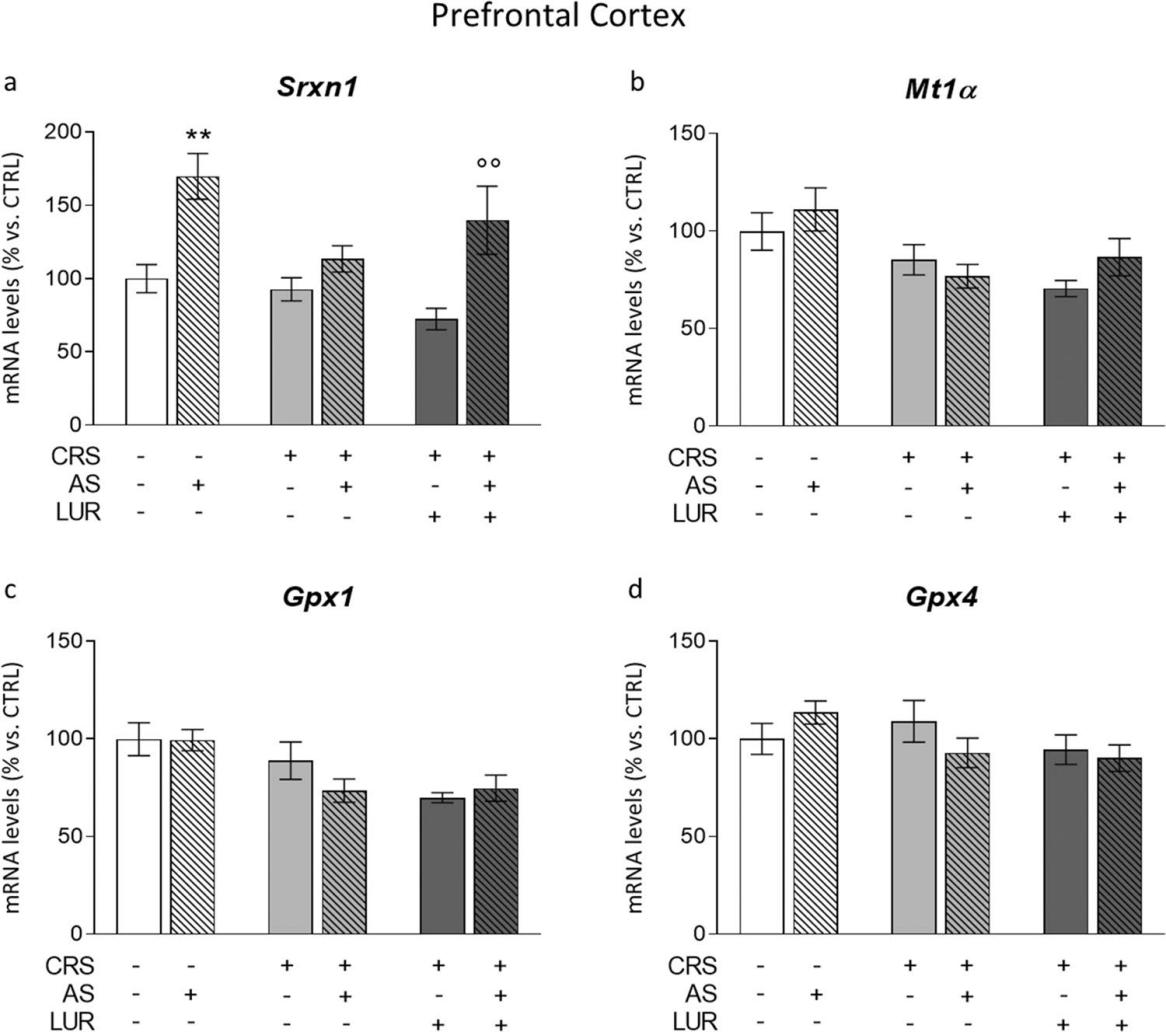
Analysis of antioxidant genes *Srxn1*, *Mt1α*, *Gpx1*, and *Gpx4* levels in the prefrontal cortex of rats exposed to acute and chronic stress and treated with lurasidone. The mRNA levels of *Srxn1* (**a**), *Mt1α* (**b**), *Gpx1* (**c**), and *Gpx4* (**d**) were analyzed in the prefrontal cortex of chronically stressed rats, treated with lurasidone and exposed to acute stress after a period of washout. The data, expressed as a percentage of control animals (CTRL, set at 100%), represent the mean ± SEM of at least 7 independent determinations. **P < 0.01 vs. CTRL; °°*P* < 0.01 vs. CRS/LUR (two-way ANOVA with Fisher’s protected LSD)

In the prefrontal cortex only *Srxn1* was modulated by the acute stress, as indicated by the two-way ANOVA (*F*_1, 39_ = 21.22; *P* < 0.0001). Specifically, the challenge induced a marked increase in the expression of the enzyme selectively in control animals (AS: + 70% vs. CTRL, *P* < 0.0001, Fig. 4a), but not in animals previously exposed to CRS. Conversely, the AS-dependent upregulation was normalized in CRS animals that were chronically treated with lurasidone (AS/CRS/LUR: + 94% vs. CRS/LUR, *P* < 0.01).

Regarding *Mt1α* (Fig. 4b), we observed a significant effect of the previous conditions (*F*_2, 47_ = 6.079; *P* = 0.0045, Fig. 4b), with a general trend to decrease in response to chronic stress. A similar expression profile was found for *Gpx1*, with a two-way ANOVA effect of the previous conditions (F_2, 40_ = 7995; *P* = 0.0012, Fig. 4c). Finally, *Gpx4* mRNA levels were not altered by our paradigm in any experimental group (Fig. 4d).

### Z-activation analyses of the antioxidant response in rats exposed to an acute challenge three weeks after chronic stress exposure and lurasidone treatment

**Fig. 5:**
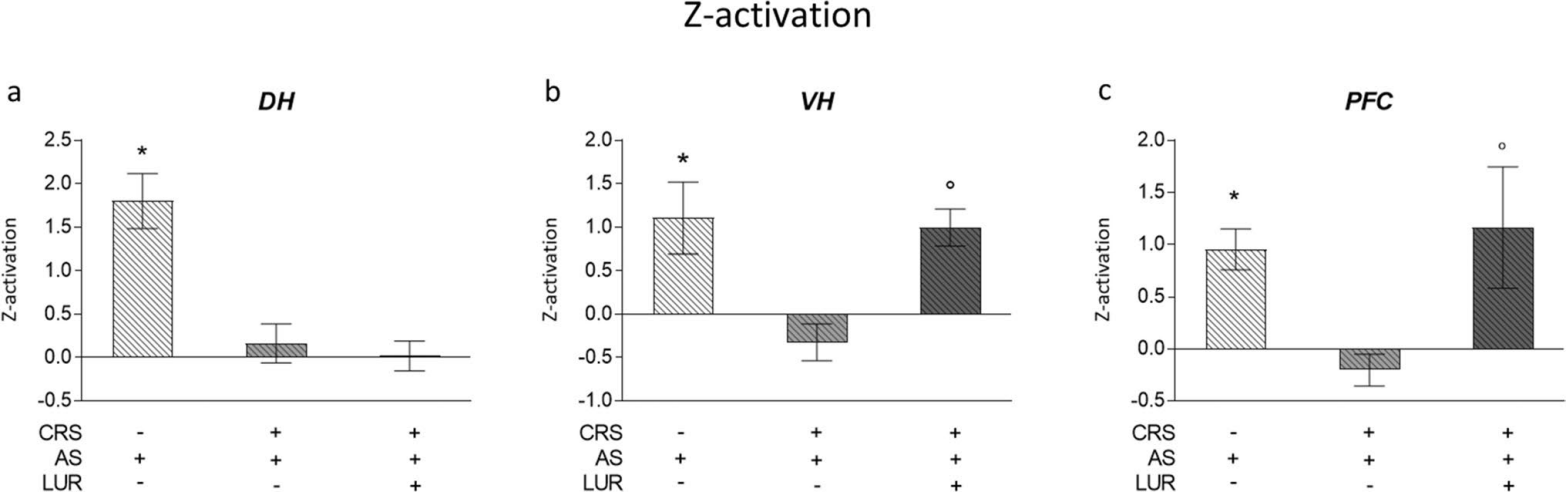
Analysis of the antioxidant z-activation to evaluate the regional antioxidant response after acute and chronic stress and lurasidone treatment. The data of each antioxidant gene were normalized using the Z score method on the respective control groups. Individual Z scores were then averaged to obtain the z-activation for each animal. The data represent the mean ± SEM of at least 7 independent determinations. *P < 0.05 vs. CTRL; °*P* < 0.05 vs. CRS/LUR (two-way ANOVA with Fisher’s protected LSD)

In order to highlight the regional differences in the acute antioxidant stress response, we evaluated the global antioxidant score (z-activation) for each brain region analyzed, by calculating the sum of the normalized z-score of each antioxidant gene for every subject. This mathematical tool is useful to integrate different interrelated gene expression data, in order to understand what are the areas that are mostly affected or responsive in an experimental setting (Marchisella et al. 2020). The average antioxidant z-activation for the experimental groups exposed to acute stress is shown in Fig. 5 as the delta activation with respect to the relative non-acutely stressed control.

In the dorsal hippocampus (Fig. 5a), acute stress produced an overall increase in the z-antioxidant score (AS: *P* < 0.05 vs. CTRL) that was not observed in CRS animals. In this brain region, the pharmacological treatment did not produce any significant modulation.

In the ventral hippocampus (Fig. 5b), we found a global antioxidant response after acute stress exposure, as indicated by the significant increased z-activation (AS: *P* < 0.05 vs. CTRL). This modulation was completely blunted in CRS/AS animals and was instead preserved in animals treated with lurasidone (CRS/LUR/AS: *P* < 0.05 vs. CRS/LUR).

Finally, also in the prefrontal cortex (Fig. 5c), the z-activation revealed a significant antioxidant response to the acute stress (AS: *P* < 0.05 vs. CTRL), that disappeared in CRS animals. Concerning the pharmacological treatment, lurasidone administration was able to restore the proper acute responsiveness of CRS/LUR/AS animals (CRS/LUR/AS: *P* < 0.05 vs. CRS/LUR).

The antioxidant z-activation showed an overall increase following the acute challenge. However, even though chronic stress per se did not alter the antioxidant genes’ expression, the acute response observed in control animals was no longer present in CRS animals and partially restored by pharmacological intervention.

## Discussion

In the present study, we demonstrated that acute stress triggers an antioxidant response that, despite a washout period, is impaired by a previous chronic adverse experience and is restored by the antipsychotic lurasidone in a brain region-specific manner.

The processes set in motion by the brain after an acute challenge represent a compensatory or adaptive response aimed at restoring homeostasis. In particular, among the molecular events occurring during—and in the short time after—acute stress, HPA axis activation plays a crucial role. This activation is accompanied by an increased energy demand and expenditure (Spencer et al. 2012; Yuen et al. 2009), which causes ROS and free radicals production leading to oxidative stress. As a consequence, the cerebral antioxidant machinery is triggered to re-establish the oxidative balance (Şahin and Gümüşlü, 2004). In line with this scenario, we observed an overall upregulation in the transcription of antioxidant enzymes and scavengers. In particular, the increase of the oxidant scavenger *sulfiredoxin-1* was observed in all the cerebral regions examined. *Srxn1* acts as a reducing agent of hyper-oxidized peroxiredoxins, reactivating them to function as reducers of H_2_O_2_ to H_2_O, thus suppressing oxidative stress (Sunico et al. 2016). Moreover, *Srxn1* is one of the genes regulated by the transcription factor Nrf2. The Nrf2 pathway is crucial in maintaining oxidative balance at the central level and its impairment has also been linked to psychiatric disorders such as major depression. Indeed, reduced levels of Nrf2 have been observed in the serum (Hamed et al. 2020) and in the prefrontal cortex of depressed subjects (Martín-Hernández et al. 2018), as well as in the chronic mild stress preclinical model of depression (Rossetti et al. 2018). These alterations were also paralleled by increased oxidative stress markers.

Concerning the other targets analyzed, we observed a region-specific effect of acute stress. Indeed, *Mt1α* is activated only in the hippocampus, in agreement with the strong increase of this protein found in the same brain region of rats exposed to a similar stress paradigm (Chen et al. 2018). Differently, the increase of *Gpx4* was restricted to the dorsal portion of this area, whereas the expression of *Gpx1* is not modulated by the acute stress. It has to be noted that in the dorsal hippocampus *Gpx1* was the only target modulated by chronic stress exposure, with a significant reduction despite the 3 weeks of recovery period. This peculiar modulation could be due to a different transcription kinetic of this specific enzyme, with a late and persistent alteration by stress.

The observed antioxidant response was significantly blunted following exposure to chronic stress, even after a washout period, suggesting that an unbalance of redox mechanisms may represent a long-lasting consequence of the stressful experience and may impair the ability of the brain to cope with challenging conditions. In line with our data, it has been recently demonstrated that the behavioral vulnerability to a depressive-like phenotype in a double-hit stress paradigm was related to antioxidant failure, associated with a reduced activation of Nrf2. Interestingly, the Nrf2 antioxidant pathway may be activated by the brain-derived neurotrophic factor (BDNF), one of the major mediators of neuroplasticity involved in stress response and stress-related psychiatric disorders (Bouvier et al. 2017). Accordingly, the lack of antioxidant acute responsiveness found in our study in animals exposed to chronic stress may be due—at least in part—to a deficiency in BDNF expression and/or function. To support this hypothesis, we recently found an impairment of BDNF signaling in the rat prefrontal cortex following the same double-hit stress paradigm (Brivio, et al. 2020a, b). Despite BDNF deficit, another candidate that may contribute to the observed antioxidant failure is the immune/inflammatory system, whose role in both stress response and psychiatric diseases is well recognized (Slavich and Irwin 2014). Indeed, it has been observed that exposure to a double-hit stress paradigm increases microglia expressing the receptor for advanced glycation end products (RAGE), whose activation is a trigger for oxidative stress and neuroinflammation (Franklin et al. 2018).

To note, we did not observe any modulation by chronic stress per se, a lack of effect that could be a consequence of the washout period. Indeed, although a reduced production and activity of antioxidant enzymes were found following the exposure to different paradigms of chronic stress (Akpinar et al. 2008; Che et al. 2015; Omar and Tash 2017), it has been demonstrated that both hippocampus and prefrontal cortex are able to partially recover after a period of washout from the adverse experience (Hoffman et al. 2011; Moench et al. 2018).

The observed impairment in the antioxidant acute responsiveness was rescued, at least in part, by the treatment with the antipsychotic lurasidone. This effect was particularly marked in the ventral hippocampus and, although to a lesser extent, in the prefrontal cortex, as indicated by the Z-activation analysis. The anatomical selectivity of this effect is particularly interesting, considering the important role played by the connections between the ventral hippocampus and prefrontal cortex in the modulation of emotional response and affective state (Fanselow & Dong 2010). Furthermore, the prefrontal cortex plays a key role in the responsiveness to antipsychotic treatment in particular for cognitive and emotional dysfunctions that have been associated with a dysregulated dopaminergic and serotonergic system (Artigas 2010). Considering the therapeutic properties of lurasidone in patients with unipolar and bipolar depression (Sanford and Dhillon 2015; Tsai et al. 2017), and the contribution of the ventral hippocampus and prefrontal cortex in the control of the emotional state, our data provide a new insight on the molecular mechanisms that may contribute to the therapeutic effect of the drug in psychiatric stress-related disorders.

Furthermore, this work is in line with the idea that antipsychotics might regulate alterations in the oxidative balance as part of their common pharmacological activity. Indeed, similar properties of what we observed were also described for other drugs, including blonanserin (Paladini et al. 2021), risperidone (Stojković et al. 2012) and paliperidone (Macdowell et al. 2016), clozapine (Möller et al. 2011), quetiapine (Han et al. 2015), and lurasidone itself in a different stress paradigm (Rossetti et al. 2018). We might hypothesize that a potential mechanism underlying lurasidone’s antioxidant ability could involve the blockade of 5-HT2A receptor, known to be associated with decreased oxidative stress and increased availability of antioxidant enzymes (Kaur and Krishan 2020; Pang et al. 2016). In addition, lurasidone acts as a partial agonist of the 5-HT1A receptor, whose activation has been linked—although in a different context—to the transcriptional induction of antioxidant genes (Biswal et al. 2015). In addition, given the high binding affinity of lurasidone to serotonin 5-HT7 receptor (Ishibashi et al. 2010), we cannot exclude that this drug-receptor interaction might contribute to the antioxidant properties observed. The signaling pathways activated by the 5-HT7 receptor are well known, as well as their potential long-term impact on some adaptive mechanisms (Okubo et al. 2021); however, a direct link to modulation of oxidative balance within the brain is still to be investigated and further studies are demanded to clarify this issue.

Nevertheless, it is difficult to establish the exact mechanisms through which this drug modulates the oxidative balance in our study, given the complexity of its receptor profile on one hand, and our experimental paradigm on the other, which is characterized by a washout period between the pharmacological treatment and the molecular analyses. Indeed, we believe that the observed molecular effects are certainly triggered by the drug-receptor interaction, but are also the result of complex adaptive mechanisms occurring later at the subcellular level through a cross-talk with different pathways and systems involving, among many others, neurotransmitters, hormones, and inflammatory mediators. In this perspective, we now provide evidence for lurasidone ability in ameliorating the long-lasting consequences of chronic stress on the antioxidant machinery even after the cessation of the treatment.

To conclude, it should be considered the limitation of a single 1-h sacrifice timepoint. Indeed, each different enzyme possesses a peculiar kinetic, and it is therefore difficult to discriminate if the modulation observed is the result of the timepoint choice or of a target-specific action of stress and/or lurasidone. However, the evaluation of the different enzymes outlines a global picture of the CNS antioxidant response to stress, supporting their potential role as a target of pharmacological intervention.

## Data Availability

The data that support the findings of the present study are available from the corresponding author upon reasonable request.
